# High affinity anti-TIM-3 and anti-KIR monoclonal antibodies cloned from healthy human individuals

**DOI:** 10.1371/journal.pone.0181464

**Published:** 2017-07-19

**Authors:** Stefan Ryser, Angeles Estellés, Edgar Tenorio, Lawrence M. Kauvar, Mikhail L. Gishizky

**Affiliations:** Trellis Bioscience LLC, Menlo Park, California, United States of America; Duke University School of Medicine, UNITED STATES

## Abstract

We report here the cloning of native high affinity anti-TIM-3 and anti-KIR IgG monoclonal antibodies (mAbs) from peripheral blood mononuclear cells (PBMC) of healthy human donors. The cells that express these mAbs are rare, present at a frequency of less than one per 10^5^ memory B-cells. Using our proprietary multiplexed screening and cloning technology CellSpot™ we assessed the presence of memory B-cells reactive to foreign and endogenous disease-associated antigens within the same individual. When comparing the frequencies of antigen-specific memory B-cells analyzed in over 20 screening campaigns, we found a strong correlation of the presence of anti-TIM-3 memory B-cells with memory B-cells expressing mAbs against three disease-associated antigens: (i) bacterial DNABII proteins that are a marker for Gram negative and Gram positive bacterial infections, (ii) hemagglutinin (HA) of influenza virus and (iii) the extracellular domain of anaplastic lymphoma kinase (ALK). One of the native anti-KIR mAbs has similar characteristics as lirilumab, an anti-KIR mAb derived from immunization of humanized transgenic mice that is in ongoing clinical trials. It is interesting to speculate that these native anti-TIM-3 and anti-KIR antibodies may function as natural regulatory antibodies, analogous to the pharmacological use in cancer treatment of engineered antibodies against the same targets. Further characterization studies are needed to define the mechanisms through which these native antibodies may function in healthy and disease conditions.

## Introduction

The immune surveillance concept asserts that a healthy individual’s immune system not only neutralizes foreign agents but also eliminates emerging tumor cells before they cause overt disease [1]. Consequently, a footprint of past antibody responses against infectious agents and emerging tumor cells exists in the memory B-cell compartment of healthy individuals. Such antibodies targeting disease associated antigens, cloned from healthy individuals, provide a particularly attractive source of therapeutics.

Trellis’s proprietary CellSpot^TM^ technology enables interrogating the memory B-cell compartment to detect IgG producing memory B cells that occur at frequencies too low to be readily exploited by other antibody discovery methods, and to clone the antibodies derived from those cells [2]. Using CellSpot we have previously cloned antibodies that bind to the gB viral glycoprotein of Human Cytomegalovirus (HCMV) [3], to hemagglutinin (HA) proteins from diverse strains of influenza A and B viruses [4], to the G glycoprotein of Respiratory Syncytial Virus (RSV) [5], and to bacterial proteins of the DNABII family implicated in biofilms [6]. For each of these targets, mAbs have been selected as therapeutic candidates and are currently in preclinical development.

As our understanding of the immune system has evolved, immune checkpoint inhibitor (ICI) proteins have become appreciated for their important physiologic role. Immune checkpoints refer to intrinsic inhibitory pathways of the immune system that are required for normal modulation of the duration and amplitude of immune responses. ICI proteins are expressed on immune cells and can function to either attenuate or augment an immune response. Elevated expression of ICI proteins has been associated with various disease conditions including cancer, auto-immunity and sepsis [7, 8, 9, 10]. Because many of the immune checkpoints are initiated by ligand–receptor interactions, they can readily be blocked by antibodies. Recently approved antibody therapies targeting inhibitory ICI proteins such as cytotoxic T lymphocyte-associated protein 4 (CTLA-4) and programmed cell death protein 1 (PD-1) and its ligand (PD-L1) have demonstrated valuable clinical benefit in the treatment of cancer [11].

In addition to using CellSpot for discovery of antibodies to infectious agents, we have found that the technology can also be used to detect and clone high affinity antibodies from memory B-cells reactive toward a variety of intrinsic antigens from healthy individuals, including ALK (anaplastic lymphoma kinase). This work stimulated our interest to determine if memory B-cells targeting ICI proteins existed in healthy human blood donors. Specifically, the extracellular domains of eight ICI proteins were used to evaluate the presence of antigen specific memory B-cells. The ICIs screened were: B7 homologue 3 (B7H3); Carcinoembryonic antigen-related cell adhesion molecule 1 (CEACAM1); Lymphocyte-activation gene 3 (LAG-3); killer cell immunoglobulin like receptor 2DL3 (KIR2DL3); Programmed cell death protein 1 (PD-1); Programmed cell death-ligand 1 (PD-L1); T-cell immunoglobulin domain and mucin domain-3 (TIM-3); and V-domain immunoglobulin (Ig)-containing suppressor of T-cell activation (VISTA). Memory B-cells targeting five of the eight ICI proteins screened were detected in healthy blood donors. The presence of specific memory B-cells varied among the individual donors, and when detected, the frequencies were considered rare, present at less than one per 10^5^ memory B-cells. The presence of these specific memory B-cells was confirmed by cloning monoclonal antibodies against the five ICI proteins.

Antibodies cloned from these memory B-cells were IgG isotypes and exhibited low nM to low pM affinity toward the specific antigen; they were qualitatively distinct from germ line IgM antibodies that are well known to include anti-self reactivities. We report here further characterization of native antibodies targeting two ICI proteins: 1) TIM-3 protein, a type 1 transmembrane protein, member of the T cell immunoglobulin and mucin domain (TIM; also known as CD366) receptor family which can regulate the function of lymphocytes and myeloid cells [12]; and 2) KIR2DL3 protein, a type 1 transmembrane protein and member of killer cell immunoglobulin-like receptors family (KIR; also known as CD158) that serves as a key regulator of human NK cell development and function [13].

MAbs targeting TIM-3 (TSR-022) and KIR (lirilumab) proteins, produced from *in vitro* somatic hypermutation of a library of human germ lines and from humanized transgenic mice, respectively, are currently being evaluated in early stage clinical trials as anti-ICI therapeutics to treat various cancers [14, 15]. Interestingly, the native antibodies we cloned from healthy humans targeting TIM-3 and KIR exhibit similar characteristics to TSR-022 and lirilumab. These native human antibodies have similar if not higher affinity toward their targets (low pM affinity) and at least one of the native anti-KIR mAbs appears to bind to a similar extracellular region on KIR as does lirilumab. Identifying high affinity antibodies targeting ICI proteins in healthy individuals suggests the intriguing possibility that these antibodies play an unanticipated physiologic role in the regulation of an immune response.

## Materials and methods

### Antigen production

The antigens used for CellSpot screening, ELISA and Octet assays were produced similarly (with exception of Flu-B hemagglutinin and TWEAK that were purchased). For the targets that were produced, genes encoding the proteins were synthesized by GeneArt (Thermo Fisher Scientific, Waltham, MA) and cloned as 6XHis tagged fusion proteins into the pTT5 expression vector (licensed from Canadian National Research Council). HEK 293 cells (Thermo Fisher Scientific, Waltham, MA) were transfected using linear PEI (Polysciences, Warrington, PA) and purification from the supernatant was performed using His60 beads (Clontech Laboratories, Mountain View, CA) according to the manufacturer recommendations. DNABII comprises bacterial Integration Host Factor (IHF) and Histone-like DNA-Binding Proteins (HU): *Staphylococcus aureus* (WP_033859538), *Pseudomonas aeruginosa* (WP_031638906), *Klebsiella pneumoniae* (WP_004143152), and *Haemophilus influenzae* (WP_005657421). Similarly, the DNA encoding the extracellular domains of ALK (ACI47591.1), TIM-3 (AAL65157.1), KIR2DL1 (ADI59746), KIR2DL2 (ACX71616), KIR2DL3 (NP_056952), KIR3DL3 (ACN38803), B7-H3 (NP_001019907), VISTA (NP_071436), PD-1 (NP_005009), PD-L1 (NP_001300958), LAG-3 (P18627), HER3 (P21860), Megakaryocytic Potentiating Factor, MPF (AAV87530) coding sequences were also produced by GeneArt. Integrity of the proteins was verified by SDS-PAGE and Coomasie Blue staining (Thermo Fisher Scientific, Waltham, MA). For TIM-3 and CEACAM1 (AAH24164), the amino terminal V domains were produced by cloning the corresponding DNA fragments in frame with the His Tag into the pTT5 vector and expressed in HEK 293 cells as previously described. Influenza B virus (B/Florida/4/2006) Hemagglutinin / HA Protein (His Tag) was purchased from Sino Biological (cat # 11053-V08H-10); and human TWEAK from EMD-Millipore (cat #GF102). Some of the KIR molecules that were only used in ELISAs were purchased from R&D systems: KIR2DL4-Fc (cat# 2238-KR-050); KIR2DS4-Fc (cat# 1847-KR-050); KIR3DL2-Fc (cat# 2878-KR-050) and KIR3DS1-Fc (cat# 4136-KR-050).

### Production of a lirilumab-similar antibody

Antibody was expressed based on published amino acid sequence for 1-7F9. The full length variable heavy and light chain DNA sequences for the lirilumab-similar mAb were synthesized by GeneArt and cloned into the pTT5 expression vector as an IgG1 isotype. Similarly, HEK 293 cells were transfected using linear PEI and purification from the supernatant was performed using Protein A (Mab Select Sure, GE Healthcare). Lirilumab is an IgG4 isotype mAb. The VH and VL germ line sequences of lirilumab are IGHV1-69*13 and IGKV3-30*15, respectively. Biacore-derived K_d_ values for lirilumab/1-7F9 were reported as 0.43nM for KIR2DL1 and 0.025nM for KIR2DL3 [16].

### CellSpot or single B-lymphocyte mAb discovery technology

Leukopaks were obtained from 27 anonymized donors under informed consent approved by Stanford’s Institutional Review Board (Stanford Blood Center; Stanford, CA). Peripheral Blood Mononuclear Cells (PBMCs) were prepared by standard methods and individual memory B-cells assayed following stimulation to proliferate and differentiate into plasma cells. A portion of the culture was allowed to secrete IgG and the footprints screened at the single cell level using a multiparameter assay that allows concurrent measurement of binding to the different targets conjugated to aldehyde groups on distinguishable fluorescent beads using sodium cyanoborohydride [2]. Lack of binding to beads coated with bovine serum albumin (BSA) was used as a specificity counterscreen. We found that the percentage of memory B-cells (CD19/CD27 positive cells) in PBMCs varied among different blood donors (0.4–4.0%). The number of memory B-cells tested in a single CellSpot experiment varied between 75,000 and 200,000 memory B-cells.

After identifying a human B-cell secreting a mAb meeting the selection criteria, the encoding mRNAs for heavy and light chains were amplified by single cell RT-PCR from sibling cells and subcloned into the previously described pTT5 vector as an IgG1. Following the same protocol as for expression of the target proteins, recombinant antibodies were produced in HEK 293 Freestyle cells by transient transfection and purified using Protein A.

### ELISA for immune checkpoint protein extracelluar domains

Binding was evaluated against purified ECDs of TIM-3, KIR2DL1, -L2 and -L3, or KIR3DL3 in an adsorption ELISA assay. The proteins were diluted in PBS and passively adsorbed on a Microlon High Binding plate (Grenier/Thermo Fisher Scientific, Waltham, MA) overnight at 4°C. TRL mAbs were diluted in blocking buffer (PBS/3% BSA) and added to the plate in serial dilutions. Horseradish peroxidase (HRP)-conjugated anti human IgG was used as detection antibody in conjunction with TMB (tetramethylbenzidine) substrate, recording optical density at 450 nm. Affinity calculations were performed using Prizm (GraphPad Software, La Jolla, CA).

ELISA for KIR2DL4-Fc, KIR2DS4-Fc, KIR3DL2-Fc and KIR3DS1-Fc were performed similarly with the exception that Horseradish peroxidase (HRP)-conjugated anti kappa and anti-lambda antibodies were used as detection antibodies instead of anti-IgG.

### Plasma ELISA

Plasma samples were obtained from anonymized donors under informed consent approved by Stanford’s Institutional Review Board (Stanford Blood Center; Stanford, CA). These donors were the same as those screened using CellSpot technology, described above. Plasma was prepared by standard methods. Adsorption ELISA was performed by coating each antigen on 96 ELISA plates as described above. Each plasma sample was diluted 1/1000 in blocking buffer and the same protocol was used as described above.

### Binding analysis for antibodies to TIM-3 and KIR2 extracellular domain

Bio-Layer Interferometry (ForteBio Octet) was performed following recommendations of the manufacturer (Pall ForteBio LLC, a division of Pall Life Sciences; Fremont, CA). Anti-human Fc biosensors were used to couple TRL6061 or TRL8605 and then either ECDs of TIM-3 or KIR2DL1 were allowed to bind in solution. After a period of wash, other antibodies were allowed to compete for binding to the antigens.

### Statistical analysis

The Pearson correlation coefficient, a measure of the strength of a linear association between two variables, was used to determine the linear association of frequency of positive antigen-specific memory B-cells determined using CellSpot for various infectious, tumor cell associated and immune check point modulator antigens. The Pearson correlation coefficient can take a range of values from +1 (positive association) to -1 (negative association). A value of 0 indicates that there is no association between the two variables. A value greater than zero indicates a positive association. Coefficients between -0.5 to -1.0 and 0.5 to 1.0 indicate a large negative or positive, respectively, strength of associate between the factors being analyzed. A p-value less than 0.05 indicates the linear association is statistically significant. For our analysis we set a cut point of N ≥ 5 and Pearson correlation coefficient ≥ 0.8 with a p-value ≤ 0.05 to define a positive significant association.

## Results

### Frequency of antigen-specific memory B-cells for various antigens

PBMCs from healthy human blood donors were screened for the presence of memory B-cells with specific reactivity toward various antigens, including eight immune checkpoint inhibitory (ICI) proteins as screening probes. The presence of memory B-cells reactive towards specific ICI proteins varied within each donor screened. Furthermore, the frequency of a memory B-cell reactive toward a specific ICI antigen within an individual donor was on average less than one per 10^5^ circulating memory B-cells (Table 1). At this frequency, detection of antigen-specific antibody in plasma by other analytical methods, such as ELISA, is very unlikely, if not impossible [17]. Therefore, it was not surprising that we could not detect antibodies toward the specific ICI proteins in the plasma of these healthy human donors using ELISA (data not shown). In contrast, memory B-cells reactive toward influenza B virus HA protein were detected in all of the screened healthy donors at a frequency of greater than 20 per 10^5^ circulating memory B-cells and anti-HA antibodies were detected in the plasma of these donors by ELISA. This high number of anti-HA memory B-cells likely reflects the broad and repeated exposure of most people to influenza B virus or to influenza vaccines that contain this antigen (Table 1).

**Table 1:** The mean frequency of antigen specific memory B-cells for various antigens using CellSpot. Antigens are listed in order of increasing mean frequency of reactive memory B-cells within positive healthy donors.

Antigen	Number of donors screened	Number of positive donors	Mean frequency of antigen specific memory B-cells per 10^5^ memory B-cells in screened donors
CEACAM1	15	10	0.20
B7-H3	22	9	0.20
TIM-3	20	14	0.64
EPO	8	5	0.74
KIR2DL3	22	19	1.08
LAG-3	14	13	1.20
ALK	11	11	1.75
HER3	5	5	4.10
Biofilm (DNABII)	11	11	4.81
MPF	5	5	7.36
TWEAK	5	5	14.06
Influenza B (HA)	14	14	22.56

### Cloning anti-TIM-3 antibodies from healthy individuals

Using our proprietary CellSpot technology and the entire extracellular domain (ECD) of TIM-3 as the antigen, native human anti-TIM-3 antibodies were cloned from four different healthy donors (TRL6042, TRL6061, TRL6099 and TRL6120). These antibodies originally were of IgG1 or IgG3 subtype. Those of IgG3 isotype used identical V gene segments for both heavy and light chains, even though they differed in sequence and the length of the CDR regions (Table 2, amino acid sequences not shown). All four mAbs were cloned and expressed as IgG1 antibodies, and are capable of binding to the soluble ECD, and at least two of them (TRL6042 and TRL6061) have been shown to bind to the membrane bound form of TIM-3 (S4 Fig). Affinity of each antibody was estimated by ELISA, and ranged from 28 nM to 0.005 nM for the soluble ECD form of TIM-3 (S1 Fig). Binding studies determined that all four antibodies appear to bind to proximal or overlapping epitopes within the mucin domain of TIM-3 (Table 3). An ELISA using the amino terminal V-region of TIM-3 gave no positive signal, confirming that the TRL antibodies bound to the mucin domain (data not shown).

**Table 2:** Native anti-TIM-3 antibodies cloned from healthy human donors. Midpoint of ELISA binding curve was used to estimate the affinity as biosensor values are unreliable for very high affinity mAbs (S1 Fig). All TRL mAbs regardless of their original isotype were cloned and tested as IgG1 mAbs.

Monoclonal antibody	Donor	VH germ line	VL germ line	Original Isotype	TIM-3 ELISA K_d_ (nM)
TRL6061	SBC207	IGHV3-33*01	IGLV3-10*01	IgG3	0.005
TRL6120	SBC238	IGHV3-33*01	IGLV3-10*01	IgG3	28
TRL6099	SBC254	IGHV3-38*01	IGLV1-44*01	IgG1	0.011
TRL6042	SBC236	IGHV5-51*01	IGKV1-9*01	IgG1	5

**Table 3:** Bio-Layer Interferometry (ForteBio Octet) was used to determine the binding of TRL antibodies to the extracellular domain of TIM-3. TIM-3 binding sites of the four mAbs are in close proximity since binding of the high affinity TRL6061 precludes binding of the three other antibodies to TIM3. TRL308 is an isotype control.

Antigen Binding (Octet)				
Sensor	1^st^ antibody	Antigen	2^nd^ antibody	Results
Anti-human Fc	TRL6061	TIM-3	TRL6042	No binding
	TRL6061	TIM-3	TRL6099	No binding
	TRL6061	TIM-3	TRL6120	No binding
	TRL6061	TIM-3	TRL308	No binding

### Cloning anti-KIR antibodies from healthy donors

Four anti-KIR antibodies (TRL8504, TRL8507, TRL8605 and TRL8608) were cloned using the ECD of KIR2DL3 protein as a probe in CellSpot. These antibodies were obtained from different healthy blood donors and were originally of IgG1 or IgG3 subtype, but cloned and expressed as IgG1 antibodies (Table 4). Affinity of each antibody was estimated by adsorption ELISA, and ranged from 79 to 0.005 nM for the soluble form of KIR2DL3 (S2 Fig).

**Table 4:** Native anti-KIR antibodies cloned from healthy human donors. All TRL mAbs regardless of their original isotype were cloned and tested as IgG1 mAbs (S2 Fig).

Monoclonal antibody	Donor	VH germ line	VL germ line	Original IgG subtype	KIR2DL3 ELISA K_d_ (nM)
TRL8504	SBC246	IGHV3-30*01	IGKV4-1*01	IgG1	38
TRL8507	SBC207	IGHV3-30*03	IGLV3-25*03	IgG3	0.1
TRL8608	SBC243	IGHV1-46*03	IGKV4-1*01	IgG3	79
TRL8605	SBC237	IGHV3-30*04	IGLV3-25*04	IgG1	0.005

The specificity of the KIR antibodies was evaluated by determining their binding affinity to other KIR proteins using ELISA (Table 5). The KIR antibodies varied in their binding affinity to KIR2DL1, -L2 and -L3 proteins. For example, antibody TRL8605 bound to KIR2DL1, -L2 and -L3 proteins with sub-nM affinity. In contrast, antibody TRL8507 bound KIR2DL2 and -L3 proteins and did not bind to KIR2DL1. Comparison of binding characteristics towards the different KIR proteins for the native antibodies to that of a lirilumab-similar mAb revealed that TRL8605 has a very similar binding pattern as lirilumab-similar mAb (Table 5). In addition, a binding analysis of these antibodies toward KIR2DL3 suggests that TRL8605 and lirilumab-similar antibody bind to proximal or overlapping epitopes (S3 Fig).

**Table 5:** Native anti-KIR antibodies cloned from healthy human donors were tested for cross reactivity with various members of the KIR family. The midpoint of ELISA binding curve was used to estimate the affinity as biosensor values are unreliable for very high affinity mAbs [18].

		ELISA K_d_ (nM)							
Monoclonal antibody	KIR2DL1	KIR2DL2	KIR2DL3	KIR2DL4	KIR2DS4	KIR3DL1	KIR3DL2	KIR3DL3	KIR3DS1
TRL8504	NB	12	38	NB	55	10	NB	NB	54
TRL8507	NB	40	0.1	NB	NB	NB	67	NB	NB
TRL8608	1100	1800	79	2000	NT	1900	NB	NB	1800
TRL8605	0.026	0.018	0.005	NB	2.6	NB	NB	NB	NB
Lirilumab-similar mAb	0.05	0.06	0.02	NB	20	NB	NB	NB	NB

NB: no binding observed; NT: not tested.

### Correlation among frequencies of memory B-cell to various antigens within individual donors

A survey of PBMCs from 27 healthy human blood donors revealed wide variation in the presence and frequency of memory B-cells reactive toward our sampling of foreign and endogenous human antigens (Table 6). While memory B-cells reactive to influenza B HA and DNABII were present in all individuals screened, memory B-cells reactive to ICI proteins were present in some, but not all donors (Tables 1 and 6). In addition the frequency of antigen specific memory B-cells toward different antigens varied widely within an individual.

**Table 6:** The mean frequency of antigen-specific memory B-cells per 10^5^ total memory B-cells for twelve different antigens from 27 different donors using CellSpot.

	Immune checkpoint inhibitory proteins					Infectious disease antigens		Tumor-associated antigens				Hormone
Donor	B7-H3	CEACAM1	LAG-3	KIR2DL3	TIM-3	Biofilm	Influenza B	ALK	HER3	MPF	TWEAK	EPO
						(DNABII)	(HA)					
SBC207	0.2			0.9	0.3	1.7	4.5	1.2				
SBC210	0	0.2	0.3		0.1	2.9	9.1	0.8	1		5.8	0.2
SBC222					0	5.3		1.6				0
SBC223	0			0	2.9	10.8	83.3	6.3		9.9	35.4	1.3
SBC224	0	0.3	0.4	0.2	0							
SBC227	0	0	1.7	0.4								
SBC230	0			0.9	0.6	5.6	24.1	1.1	7.7	6.8	11.1	0
SBC232	0	0	0.2	0.9								
SBC234	0.9	0.1	1.5	1.1			17.8					
SBC235	0.3	1.1	2.7	1.6	0.9							
SBC236					0.4	5.1		1	2	4.4		1
SBC237	0	0.1	1	0.8			31.5					
SBC238	0.4	0	0.9	0.3	2.6							
SBC240	0.8			0.6	1.6	6		2.6				
SBC241	0.3	0.5		1.6	0	4	23.3	0.3	5.5	7.4		1.3
SBC242						4.3		0.5				
SBC243	0	0	2.1	0.8	0		34					
SBC246	0.1			1.5	0.3	3.6	28.6	2.3				0
SBC247	0	0.5	2.2	0.9								
SBC248	1.4			0	0.7	3.6	24.6	1.5	4.3	8.3	12.5	2.1
SBC249	0	0.1	0.5	0	0.2		12.5					
SBC251	0			1.4	0							
SBC253		0	1.4									
SBC254	0	0	0.2	0.1	0.3		9.6					
SBC255	0	0.1	1.7	1	0.3		6.9					
SBC256				1	0		6.1					
SBC258	0.1			7.8	1.6						5.5	

(blank = not tested or fewer than 10^5^ memory B-cells were screened).

To determine whether there was an association between the presence and frequency of particular antigen reactive memory B-cells within an individual, a bivariate statistical analysis was conducted. When comparing the data set of the frequencies for positive memory B-cells toward each antigen, we found a statistically significant correlation (p<0.05) between frequency of memory B-cells reactive to TIM-3 and those reactive to ALK, bacterial biofilm DNABII proteins, and influenza B (Table 7). However, there appeared to be no correlation between the frequency of any of anti-TIM-3, bacterial biofilm proteins, influenza B or ALK reactive B-cells when compared to other antigens, such as, HER3, MPF, TWEAK, KIR2DL3, B7-H3, CEACAM1, LAG-3 or EPO (erythropoietin).

**Table 7:** Pearson correlation matrix. The data demonstrate a strong positive association among the frequencies of positive memory B-cells for TIM-3), and infectious disease agents (biofilm, influenza B) as well as tumor-associated antigen (ALK).

		B7-H3	CEACAM1	LAG-3	KIR2DL3	TIM-3	BIOFILM	INFB	ALK	HER-3	MPF	TWEAK	EPO
B7-H3	Correlation	1	0.155	0.228	-0.1	0.219	-0.166	-0.075	-0.135	-0.034	0.026	-0.1	0.771
	p-value		0.597	0.454	0.666	0.399	0.695	0.809	0.75	0.966	0.974	0.873	0.073
	N	22	14	13	21	17	8	13	8	4	4	5	6
CEACAM1	Correlation	0.155	1	0.534^*^	0.630^*^	-0.027	1.000^**^	0.038	1.000^**^	1.000^**^	.^c^	.^c^	1.000^**^
	p-value	0.597		0.049	0.021	0.945		0.929					
	N	14	15	14	13	9	2	8	2	2	1	1	2
LAG-3	Correlation	0.228	0.534^*^	1	0.714^**^	0.109	.^c^	0.518	.^c^	.^c^	.^c^	.^c^	.^c^
	p-value	0.454	0.049		0.009	0.798		0.234					
	N	13	14	14	12	8	1	7	1	1	0	1	1
KIR2DL3	Correlation	-0.1	0.630^*^	0.714^**^	1	0.113	-0.515	-0.271	-0.588	0.415	-0.729	-0.585	-0.588
	p-value	0.666	0.021	0.009		0.665	0.237	0.37	0.165	0.728	0.271	0.415	0.297
	N	21	13	12	22	17	7	13	7	3	4	4	5
TIM-3	Correlation	0.219	-0.027	0.109	0.113	1	0.850^**^	0.870^**^	0.921^**^	0.35	0.705	0.818	0.369
	p-value	0.399	0.945	0.798	0.665		0.002	<0.001	<0.001	0.563	0.184	0.091	0.369
	N	17	9	8	17	20	10	12	10	5	5	5	8
BIOFILM	Correlation	-0.166	1.000^**^	.^c^	-0.515	0.850^**^	1	0.963^**^	0.828^**^	0.572	0.543	0.963^*^	0.166
	p-value	0.695			0.237	0.002		<0.001	0.002	0.314	0.345	0.037	0.694
	N	8	2	1	7	10	11	7	11	5	5	4	8
INFB	Correlation	-0.075	0.038	0.518	-0.271	0.870^**^	0.963^**^	1	0.932^**^	0.854	0.893	0.999^**^	0.304
	p-value	0.809	0.929	0.234	0.37	<0.001	<0.001		0.002	0.146	0.107	0.001	0.558
	N	13	8	7	13	12	7	14	7	4	4	4	6
ALK	Correlation	-0.135	-1.000^**^	.^c^	-0.588	0.921^**^	0.828^**^	0.932^**^	1	0.021	0.706	0.992^**^	0.18
	p-value	0.75			0.165	<0.001	0.002	0.002		0.973	0.183	0.008	0.67
	N	8	2	1	7	10	11	7	11	5	5	4	8
HER-3	Correlation	-0.034	1.000^**^	.^c^	0.415	0.35	0.572	0.854	0.021	1	0.539	0.744	-0.052
	p-value	0.966			0.728	0.563	0.314	0.146	0.973		0.461	0.466	0.933
	N	4	2	1	3	5	5	4	5	5	4	3	5
MPF	Correlation	0.026	.^c^	.^c^	-0.729	0.705	0.543	0.893	0.706	0.539	1	0.899	0.386
	p-value	0.974			0.271	0.184	0.345	0.107	0.183	0.461		0.289	0.521
	N	4	1	0	4	5	5	4	5	4	5	3	5
TWEAK	Correlation	-0.1	.^c^	.^c^	-0.585	0.818	0.963^*^	0.999^**^	0.992^**^	0.744	0.899	1	0.39
	p-value	0.873			0.415	0.091	0.037	0.001	0.008	0.466	0.289		0.61
	N	5	1	1	4	5	4	4	4	3	3	5	4
EPO	Correlation	0.771	1.000^**^	.^c^	-0.588	0.369	0.166	0.304	0.18	-0.052	0.386	0.39	1
	p-value	0.073			0.297	0.369	0.694	0.558	0.67	0.933	0.521	0.61	
	N	6	2	1	5	8	8	6	8	5	5	4	8

* Correlation is significant at the 0.05 level (2-tailed)

** Correlation is significant at the 0.01 level (2-tailed)

^c^ Cannot be computed because at least one of the variables is constant

## Discussion

By leveraging our high throughput, multiplexed single B-cell discovery platform, we have found circulating memory B-cells that target the extracellular domains of five different ICI proteins in healthy human donors. Antibodies derived from these memory B-cells are highly matured, of IgG isotype, and selective toward the specific antigen with high affinity binding. Furthermore the native anti-TIM-3 and anti-KIR antibodies derived from healthy individuals exhibit characteristics similar to pharmaceutically engineered antibodies currently in clinical trials for the treatment of cancer.

The identification of naturally occurring anti-ICI antibodies in healthy human donors is surprising. Given the high affinity and selectivity of these antibodies, the fact that four anti-TIM-3 antibodies derived from four different individuals bind to the same or an overlapping epitope and that a native anti-KIR antibody has very similar binding characteristic to lirilumab, we infer that these native antibodies are not simply products of chance recombination events but rather have a physiologic function, most plausibly to regulate immune responses.

The finding that four anti-TIM-3 antibodies, each derived from a different donor, bind to the same or an overlapping epitope is intriguing and supports the hypothesis that binding to this epitope serves a biological function. The ECD of TIM-3 possesses an immunoglobulin-variable (IgV)-like amino-terminal domain and a membrane proximal mucin domain. It has been demonstrated that the IgV-like domain of TIM-3 is necessary and sufficient to bind galectin-9 and phosphatidyl serine [19]. Recently, it has been reported that TIM-3 can form heterodimers with CEACAM1 through their amino terminal Ig-V regions and that this interaction participates in immune tolerance induction [20, 21]. However, very little is known regarding the possible role of the C terminal or mucin domain on TIM-3 function. Results from ELISA studies demonstrate that the four native anti-TIM-3 antibodies bind to the full length ECD of TIM-3 but do not bind to the amino terminal IgV region suggesting these antibodies should not block TIM-3 and CEACAM1 heterodimerization. Competitive binding analysis revealed that these antibodies all bind the same or an overlapping epitope on TIM-3. Together these data indicate that the four anti-TIM-3 antibodies, each derived from a different donor, bind to a region with overlapping epitopes that is outside the amino terminal Ig-V region and supports the hypothesis that binding to this region serves a biological function.

When analyzing the CellSpot screening data, we found a statistically significant correlation between the frequency of TIM-3 positive memory B-cells and the frequencies of memory B-cells targeting bacterial biofilm proteins and HA protein of influenza B virus. This correlation implies that the role of native anti-TIM-3 human antibodies is not to eliminate TIM-3 bearing cells, but rather that anti-TIM-3 antibodies may perform a regulatory function on T-lymphocytes and other immunological cells that express TIM-3 and we therefore refer to these antibodies as regulatory antibodies. Currently we do not know the exact biological function of these anti-TIM-3 antibodies. We propose that an anti-TIM-3 antibody would function as a TIM-3 inhibitor, augmenting T-cell responses, and thereby enabling a stronger cellular and humoral immune response. While the correlation is not a proof of function, the strong association fits the hypothesis that TIM-3 antibodies inhibit TIM-3 and proportionally augment an immune response against infectious agents and emerging tumor cells.

Native anti-KIR antibodies derived from healthy donors exhibit selectivity towards distinct KIR glycoprotein family members. The KIR family is encoded by 14 polymorphic genes and distinct family members can transduce either activating or inhibitory signals that regulate NK cells. Nomenclature of KIR proteins is based upon the number of C2-type immunoglobulin-like domains in the extracellular region (2D for two domains, 3D for three domains) and by the length of the cytoplasmic domain (*L* for long-tailed receptors and *S* for short ones). The amino acid sequences of human KIR proteins are highly conserved within the extracellular domains. Because the ECD of KIR2DL3 was used in the CellSpot assay as a probe all the native mAbs cloned exhibited the highest affinity binding toward this KIR family member. However, binding toward other KIR members varied greatly which demonstrates the exquisite selectivity of these antibodies. Of particular interest is one of our anti-KIR antibodies; TRL8605 binds with 5–30 pM affinities to KIR2DL1, -L2 and -L3, which appears to be a similar binding pattern as a lirilumab-similar antibody, which binds with 20–60 pM affinities to KIR2DL1, -L2 and -L3, slightly weaker than TRL8605 (Table 5). The lirilumab-similar mAb we cloned from published sequence data has variable domains identical in sequence to the variable domains of lirilumab, an IgG4 isotype mAb in clinical development that binds KIR2DL1, -L2 and -L3 receptors. Furthermore, binding analysis of these antibodies toward KIR2DL3 suggests that TRL8605 and lirilumab-similar mAb might bind to proximal or overlapping epitopes and supports the hypothesis that the TRL8605 exhibits a natural biological function analogous to that of the engineered mAb.

In addition to anti-TIM-3 and anti-KIR mAbs we have found native human mAbs that target B7-H3, CEACAM1 and LAG-3 with affinities below 10 nM against these targets (S5–S7 Figs). However, in the same screening and cloning campaign comprising surveys of over 3.4, 1.6 and 10 million memory B-cells, we were not able to clone antibodies against PD-1, PDL-1, and VISTA, respectively. In comparison, we screened on average approximately 200,000 memory B-cells to detect a single TIM-3- or KIR2DL3-specific memory B-cell in those individuals who harbor these cells. Our inability to clone antibodies against PD-1, PDL-1 and VISTA could either be due to an issue with the antigen used in the CellSpot screening assay or may indicate that these immune checkpoint proteins are not regulated via native antibodies.

Because the mAbs we have cloned from healthy human donors appear to have arisen from a natural analogue of the pharmacological strategy that has recently shown substantial clinical utility, these TIM-3 and KIR2 antibodies offer a particularly favorable source for drug development candidates to treat patients. Further investigation of natural regulatory antibodies may reveal particularly useful combinations of anti-ICI antibodies for treatment.

## Supporting information

S1 Fig (PDF)

S2 Fig (PDF)

S3 Fig (PDF)

S4 Fig (PDF)

S5 Fig (PDF)

S6 Fig (PDF)

S7 Fig (PDF)
