# Supplementary material for: High affinity anti-TIM-3 and anti-KIR monoclonal antibodies cloned from healthy human individuals
Source: PLoS One. 2017 Jul 19;12(7):e0181464. doi: 10.1371/journal.pone.0181464 (PMC5517007; doi:10.1371/journal.pone.0181464)
Supplement: S2 Fig — (PDF) [file pone.0181464.s002.pdf]

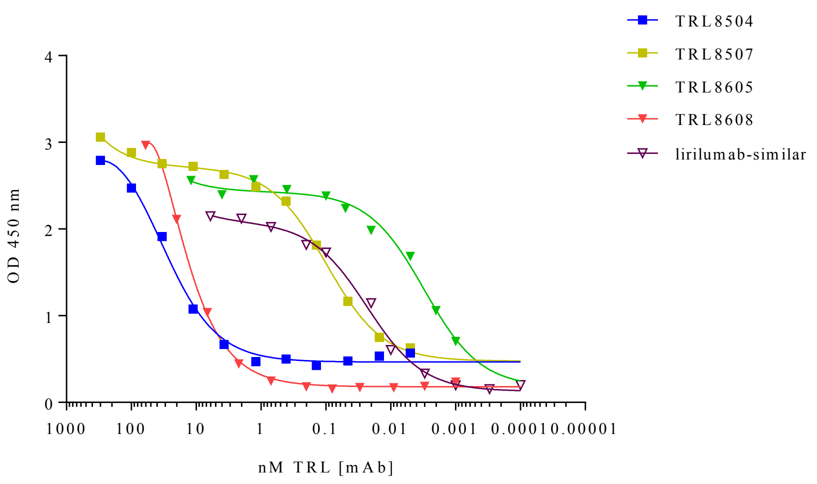

S2 Fig. Affinity determination for anti-KIR2DL3 mAbs by ELISA. ELISA binding curves were generated using the ECD of KIR2DL3 at 2 µg/mL and serial dilutions of the anti-KIR2DL3 mAbs. Midpoint of the binding curve was used to estimate the affinity (K_d) using the Prizm software.
