# Supplementary material for: High affinity anti-TIM-3 and anti-KIR monoclonal antibodies cloned from healthy human individuals
Source: PLoS One. 2017 Jul 19;12(7):e0181464. doi: 10.1371/journal.pone.0181464 (PMC5517007; doi:10.1371/journal.pone.0181464)
Supplement: S3 Fig — (PDF) [file pone.0181464.s003.pdf]

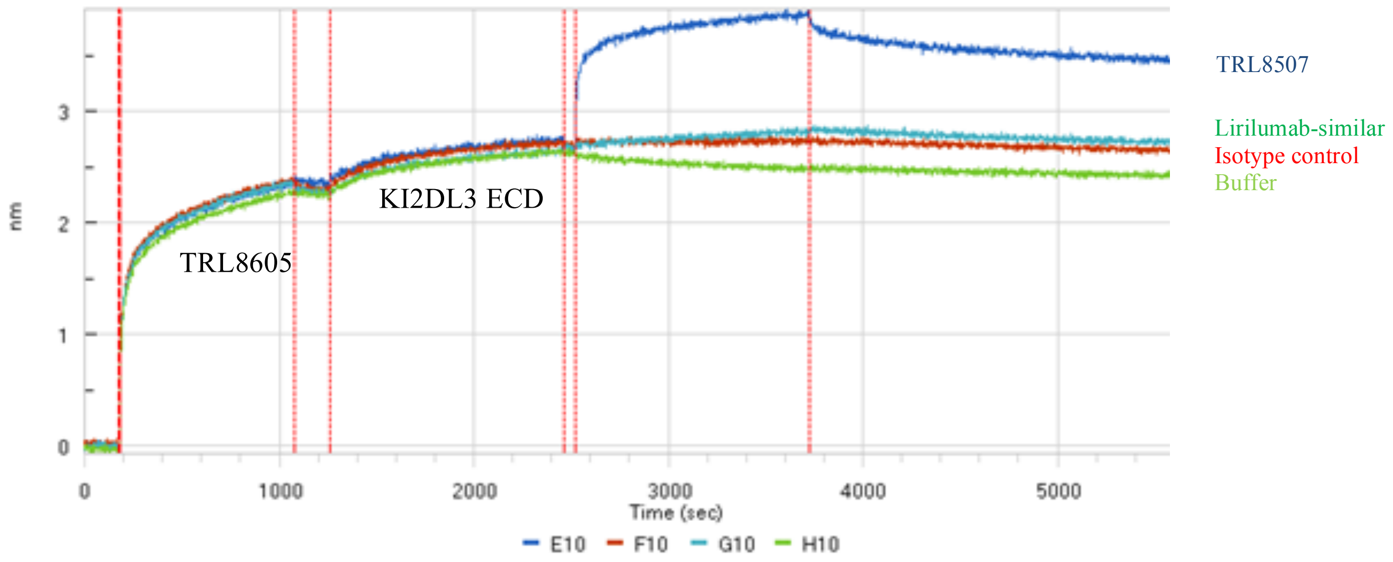

Sensor	Load 1	Load 2	Load 3
A2	TRL8605	ECD KIR2DL3	TRL8507
B2	TRL8605	ECD KIR2DL3	lirilumab-similar
C2	TRL8605	ECD KIR2DL3	Isotype control
D2	TRL8605	ECD KIR2DL3	Buffer

S3 Fig. Bio-Layer Interferometry (ForteBio Octet) was used to determine the competitive binding site for anti-KIR2DL3 mAbs. TRL8605 was first bound to the anti-Fc sensors (Load 1). KIR2DL3 added was then allowed to bind to TRL8605 (Load 2) followed by TRL8507 or lirilumab-similar (Load 3).
