# Supplementary material for: High affinity anti-TIM-3 and anti-KIR monoclonal antibodies cloned from healthy human individuals
Source: PLoS One. 2017 Jul 19;12(7):e0181464. doi: 10.1371/journal.pone.0181464 (PMC5517007; doi:10.1371/journal.pone.0181464)
Supplement: S4 Fig — (PDF) [file pone.0181464.s004.pdf]

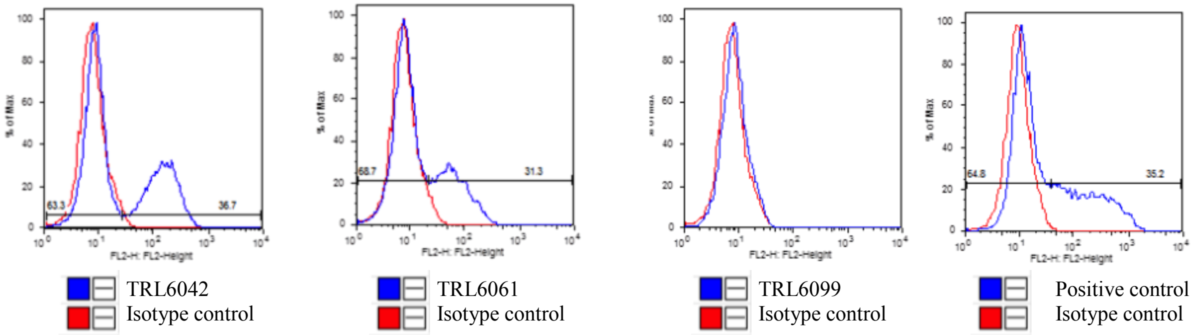

S4 Fig. Flow cytometry was used to determine the binding of anti-TIM-3 antibodies to membrane bound TIM-3. HEK293 cells were tranfected with human TIM-3 and labled with anti-TIM-3 antibodies or a mouse positive control. Overexpression of TIM-3 in transient transfection induced apoptosis in HEK293 cells. Flow cytometry was performed on Day 1 post-transfection when only 35% of cells expressed TIM-3 at high levels; hence the two peaks in flow analysis.
