# Supplementary material for: High affinity anti-TIM-3 and anti-KIR monoclonal antibodies cloned from healthy human individuals
Source: PLoS One. 2017 Jul 19;12(7):e0181464. doi: 10.1371/journal.pone.0181464 (PMC5517007; doi:10.1371/journal.pone.0181464)
Supplement: S5 Fig — (PDF) [file pone.0181464.s005.pdf]

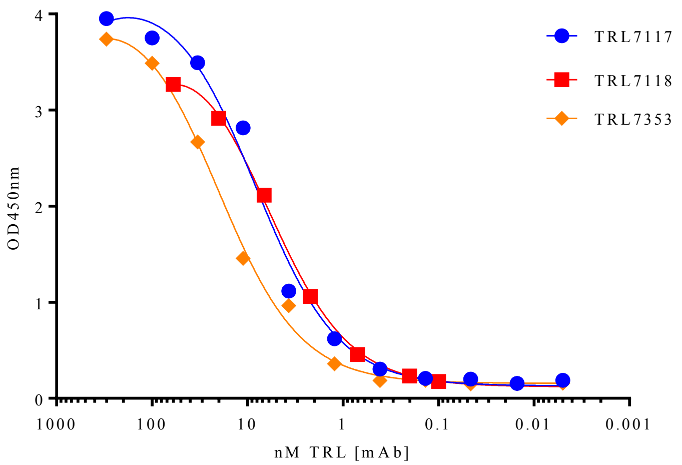

S5 Fig. Affinity determination for anti-LAG-3 mAbs by ELISA. ELISA binding curves were generated using the ECD of LAG-3 at 2 µg/mL and serial dilutions of the anti-LAG-3 mAbs. Midpoint of the binding curve was used to estimate the affinity (K_d) using the Prizm software. The calculated K_d were the following: TRL7117=8.3 nM; TRL7118=6.5 nM and TRL7353=21 nM.
