# Supplementary material for: High affinity anti-TIM-3 and anti-KIR monoclonal antibodies cloned from healthy human individuals
Source: PLoS One. 2017 Jul 19;12(7):e0181464. doi: 10.1371/journal.pone.0181464 (PMC5517007; doi:10.1371/journal.pone.0181464)
Supplement: S6 Fig — (PDF) [file pone.0181464.s006.pdf]

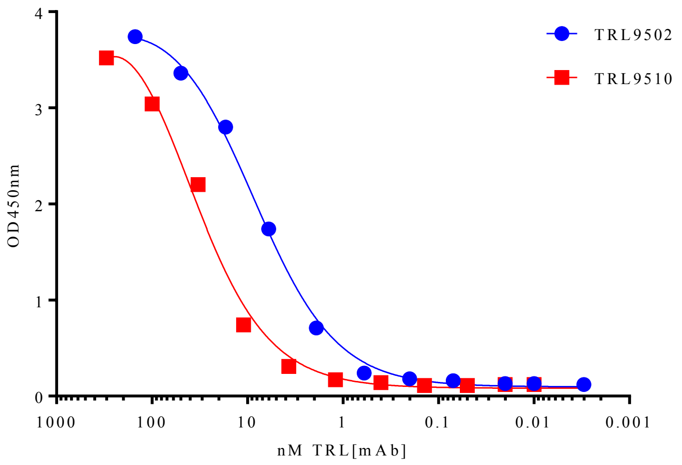

S6 Fig. Affinity determination for anti-CEACAM1 mAbs by ELISA. ELISA binding curves were generated using the ECD of CEACAM1 amino terminal domain at 2 µg/mL and serial dilutions of the anti- CEACAM1 mAbs. Midpoint of the binding curve was used to estimate the affinity (K_d) using the Prizm software. The calculated K_d were the following: TRL95027=8 nM and TRL9510=50 nM.
