# Supplementary material for: High affinity anti-TIM-3 and anti-KIR monoclonal antibodies cloned from healthy human individuals
Source: PLoS One. 2017 Jul 19;12(7):e0181464. doi: 10.1371/journal.pone.0181464 (PMC5517007; doi:10.1371/journal.pone.0181464)
Supplement: S7 Fig — (PDF) [file pone.0181464.s007.pdf]

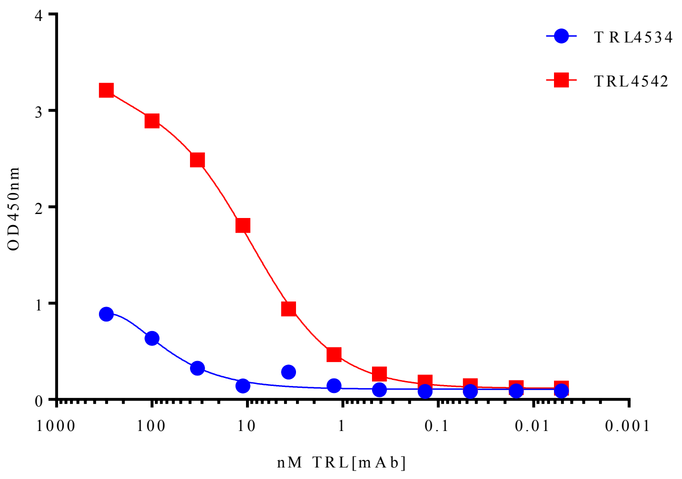

S7 Fig. Affinity determination for anti-B7-H3 mAbs by ELISA. ELISA binding curves were generated using the ECD of B7-H3 at 2 µg/mL and serial dilutions of the anti- B7-H3 mAbs. Midpoint of the binding curve was used to estimate the affinity (K_d) using the Prizm software. The calculated K_d were the following: TRL4534=377 nM and TRL4542=9 nM.
